# Changes in Cerebrospinal Fluid Concentrations of Selenium Species Induced by Tofersen Administration in Subjects with Amyotrophic Lateral Sclerosis Carrying SOD1 Gene Mutations

**DOI:** 10.1007/s12011-024-04311-4

**Published:** 2024-07-17

**Authors:** Marco Vinceti, Teresa Urbano, Tommaso Filippini, Roberta Bedin, Cecilia Simonini, Gianni Sorarù, Francesca Trojsi, Bernhard Michalke, Jessica Mandrioli

**Affiliations:** 1 CREAGEN - Environmental, Genetic, and Nutritional Epidemiology Research Center, Department of Biomedical, Metabolic, and Neural Sciences, University of Modena and Reggio Emilia, Modena, Italy; 2 Department of Epidemiology, Boston University School of Public Health, Boston, MA USA; 3 School of Public Health, University of California Berkeley, Berkeley, CA USA; 4 Center for Neurosciences and Neurotechnology, Department of Biomedical, Metabolic, and Neural Sciences, University of Modena and Reggio Emilia, Modena, Italy; 5 Neurology Unit, Modena University Hospital, Modena, Italy; 6 Department of Neurosciences, Neuromuscular Center, University of Padua, Padua, Italy; 7 Department of Advanced Medical and Surgical Sciences, MRI Research Center, Luigi Vanvitelli Campania University, Naples, Italy; 8 First Division of Neurology, University Hospital, Luigi Vanvitelli Campania University, Naples, Italy; 9 Research Unit Analytical BioGeoChemistry, Helmholtz Center Munich - German Research Center for Environmental Health, Neuherberg, Germany

**Keywords:** Amyotrophic lateral sclerosis, Selenium, Selenium compounds, SOD1, Tofersen

## Abstract

Amyotrophic lateral sclerosis (ALS) is a fatal neurodegenerative disease affecting the brain and spinal cord motor neurons. On 25 April 2023, the drug tofersen, an antisense oligonucleotide, received the US Food and Drug Administration approval for treating ALS in adults carrying mutations of the *SOD1* gene. We aimed at assessing whether cerebrospinal fluid concentrations of selenium, an element of both toxicological and nutritional interest possibly involved in disease etiology and progression, are modified by tofersen administration. We determined concentrations of selenium species by anion exchange chromatography hyphenated to inductively coupled plasma-dynamic reaction cell-mass spectrometry and overall selenium by using inductively coupled plasma sector-field mass spectrometry, at baseline and 6 months after active tofersen treatment in ten Italian ALS patients carrying the *SOD1* gene mutation. Concentrations of total selenium and many selenium species substantially increased after the intervention, particularly of inorganic (tetravalent and hexavalent) selenium and of the organic species selenomethionine and a compound co-eluting with the selenocystine standard. Overall, these findings suggest that tofersen treatment markedly alters selenium status and probably the redox status within the central nervous system, possibly due to a direct effect on neurons and/or the blood–brain barrier. Further studies are required to investigate the biological and clinical relevance of these findings and how they might relate to the pharmacological effects of the drug and to disease progression.

**Supplementary Information:**

The online version contains supplementary material available at 10.1007/s12011-024-04311-4.

## Introduction

Amyotrophic lateral sclerosis (ALS) is a generally fatal neurodegenerative disorder affecting cortical and spinal motor neurons. The familial form is diagnosed in about 5–10% of patients, while most are diagnosed with the sporadic form [1]. Approximately 2.5% of ALS cases [2] are caused by mutations in the gene encoding the protein copper/zinc superoxide dismutase 1 (SOD1) [3]. Although the precise mechanisms by which mutations in the *SOD1* gene lead to motor neuron degeneration are not fully understood, a toxic gain-of-function is probable following such mutations in *SOD1* [4, 5]. Along with other gene mutations, this could interact with yet unidentified environmental and lifestyle factors, potentially triggering ALS in individuals carrying gene mutations [1]. Unfortunately, very little evidence is available regarding these risk factors, which may include environmental chemicals such as pesticides, solvents, heavy metals, and the metalloid selenium [1, 6].

Recently, tofersen has emerged as a tentative treatment for ALS linked to *SOD1* mutations [7–10]. The drug, an intrathecally administered antisense oligonucleotide, is designed to degrade superoxide dismutase type-1 enzyme mRNA, thus reducing the synthesis and production of the protein [11, 12]. The SOD1 protein possesses antioxidant properties but is also supposed to undergo a toxic gain-of-function in mutation carriers [1, 13, 14]. Based on the results from randomized controlled trials conducted in both the USA and Europe [15, 16], on 25 April 2023, tofersen received approval from the US Food and Drug Administration for treating ALS in adults carrying the *SOD1* mutation. The Marketing Authorization Application is currently under review by the European Medical Agency, but some European countries have started administering the drug under an early access program (EAP).

The initial results of such treatment have not yet confirmed its effectiveness on the primary outcome, although there has been an indication of favorable effects on secondary endpoints [17]. A more thorough assessment of the drug efficacy and safety has yet to come, following the results from ongoing trials [7, 18]. However, the net effect of this drug on the central nervous system (CNS) and more specifically on motor neurons, including their redox status, is still unclear and is under active investigation.

Among the factors strongly influencing the redox equilibrium of biological systems including the CNS is the metalloid selenium in its different chemical forms and bound to different selenoenzymes. Selenium and selenoproteins are characterized by complex and even opposite activities in biological systems, depending on the chemical form, the amount of exposure, and the effect under investigation. For instance, selenium species have been associated to both antioxidant and pro-oxidant properties, and more generally with beneficial and adverse effects that are currently under active investigation and only partially elucidated [19–25].

Selenium administration may stimulate selenoenzyme synthesis by increasing its availability or alternatively by inducing oxidative stress and the consequent compensatory response of antioxidant enzyme synthesis [20, 22, 26]. Excess selenium exposure has been involved in ALS etiology by epidemiologic and toxicologic studies [27–32], including one case–control study performed in carriers of ALS-related gene mutations [33], though reports about ALS epidemiology in seleniferous areas are unfortunately still missing. Selenium and potentially toxic selenoproteins have also been involved in the etiology of other neurodegenerative diseases by recent human studies [34–37], further supporting the dual, opposite biological effects of this element, also depending on its dose and the chemical form [38–40] and with adverse effects occurring at unexpectedly low exposure levels [41–43].

In this study, we assess the possibility that an ongoing EAP with tofersen, carried out in Italian centers on ALS patients carrying the *SOD1* mutation, could alter the concentration of selenium species in the CNS.

## Methods

### Study Design and Population

We recruited ten adult patients affected by ALS from three major specialized Italian MND centers (Modena, Naples, and Padua), meeting all of the following four criteria: age ≥ 18 years, ALS diagnosis as established by Gold-Coast criteria, being a carrier of *SOD1* mutations (established by PCR or NGS according to the local procedure for genetic testing), and participation in a Global EAP for tofersen administration, with availability of CSF samples following standard procedures for intrathecal drug administration. Fifty percent of these patients reported a family history of the condition, and distribution of the mutations across the exons was as follows: 50% were located in exon 5, 20% in exon 1, 20% in exon 4, and 10% in exon 2. Specifically, two patients carried the E134del mutation, two had the I150T mutation, and the remaining mutations identified in the study participants were A5V, A96T, G11E, D91A, L145F, and E41G (Supplemental Table S1). EAP tofersen intervention in patients with SOD 1-ALS was approved for ten patients followed in the ALS Centers of Modena (Comitato Etico Area Vasta Emilia Nord, file numbers: 229/2021, 230/2021, 231/2021, 232/2021, and 205/2022), Padua and Naples, (file numbers: 0032241/i date: 11 Nov 2021; 0032238/i date: 11 Nov 2021; 0032228/i date: 11 Nov 2021) to record efficacy and safety from the patients enrolled in the trial. Overall, 100 mg of the drug were administered via intrathecal (IT) injection by LP with a loading period consisting of 3 doses 14 days apart from one another, and maintenance doses every 28 days thereafter. End of treatment has not been planned so far.

During scheduled visits, patients underwent clinical examination including assessment of ALSFRS-R score, FVC, ALSAQ-40 questionnaire, laboratory safety assessments, and CSF analysis with determination of proteins, glucose, cell count, and biomarkers. We also collected demographic and clinical variables, including sex; age at onset; diagnostic latency; family history for ALS and/or FTD; site of onset (bulbar, upper limb or lower limb, respiratory); phenotype (classic, bulbar, upper motor neuron predominant, flail arm, flail leg, and respiratory ALS); anthropometric measures such as weight, height, and body mass index (BMI) at diagnosis; and clinical data such as time to gastrostomy, non-invasive, or invasive ventilation. During the study period, the participants did not receive any dietary supplements containing selenium, while all of them received the drug riluzole.

### Sample Collection and Analytical Determinations

For each patient, we collected two CSF samples, one right before starting tofersen treatment and the second after 6 months of tofersen administration. Each sample was collected in the morning in fasting subjects undergoing lumbar puncture according to standard clinical and operating procedures. Each sample was received and handled by the UNIMORE Neuroimmunology Laboratory within 30 min from the collection and was centrifuged at 1300 × g for 10 min at controlled room temperature. After centrifugation, samples were stored in polypropylene sterile tubes at − 80 °C awaiting testing. Once we collected all samples, they were then transported deep-frozen in dry ice by air courier to the Analytical BioGeoChemistry laboratory in Germany.

We determined neurofilament light chain (NfL) concentrations in the CSF using automated next-generation ELISA (Ella Simple Plex assay technology, BioTechne, ProteinSimple), as previously described [44]. In this setting, samples run through a channel composed of three glass nano reactors (GNRs) coated with a capture antibody. Samples were automatically read in triplicate and loaded into the cartridge with a 1:2 dilution, evaluating intra-assay and inter-assay variability [45].

For selenium speciation analysis, we used the hyphenated system from Perkin Elmer (Rodgau, Germany) comprising a NexSAR gradient HPLC pump, auto-sampler, and NexION 300 D ICP-DRC-MS, completely controlled through the Clarity software, and the anion exchange-separation column for species separation (AG-11 + AS-11 from Thermo Dionex, Idstein, Germany). The sample volume amounted to 50 µL. The chromatography settings were as follows: Eluent A, 10 mM Tris-HAc and 5% MeOH, pH 8.0; and eluent B, 50 mM Na_2_CO_3_, 20 mM NH_4_Ac, 5% MeOH, pH 8.0. The gradient elution expressed as a percent was as follows: eluent A, 0–2 min, 100–80%; 2–8 min, 80–45%; 8–10.5 min, 45–0%; 10.5–14 min, 0%; and 14–16 min, 100%. The flow rate was 0.80 mL/min. The experimental settings for ICP-DRC-MS were as follows: radio frequency power, 1250 W; plasma gas flow, 15 L Ar/min; auxiliary gas flow, 1.05 L Ar/min; nebulizer gas flow, 0.92 L Ar/min, daily optimized; dwell time, 300 ms; ions monitored, ^77^Se, ^78^Se, and ^80^Se; DRC reaction gas, CH_4_ reaction at 0.58 mL/min; and DRC rejection parameter *q*, 0.6. We determined the concentrations of the following selenium species: selenite (Se-(IV)), selenate (Se-(VI)), selenomethionine-bound selenium (Se-Met), selenocystine-bound selenium (Se-Cys_2_), thioredoxin reductase-bound selenium (Se-TXNRD), glutathione-peroxidase-bound selenium (Se-GPX), selenoprotein P-bound selenium (Se-SELENOP), and human serum albumin-bound selenium (Se-HSA). Since SELENOP is not commercially available, we purified SELENOP from human serum applying a method based on references [46, 47], further modified and detailed in [48], using a Heparin-affinity column (Amersham, GE Healthcare Europe GmbH, Munich, Germany). SELENOP was collected under UV 280 nm monitoring from respective peak fraction. The SELENOP fraction was preconcentrated by freeze drying, re-dissolved in 1 mL of 10 mM Tris–HCl buffer, pH 7.2, and Se was determined by ICP-DRC-MS. For verification, an aliquot was subject to a mass-calibrated SEC column. The observed single Se and UV peak corresponded to the elution volume calculated for 60 kDa, which fits to literature data [49]. The remaining part of the SELENOP fraction was aliquoted for single-use standard fractions, which were shock-frozen in N_2_liq and stored deep-frozen until use. Properly stored SELENOP laboratory-made standards resulted in a single peak signal when analyzed by SAX-ICP-DRC-MS. Human serum albumin (HSA) was prepared at a concentration of 1000 mg/L. Preparation of Se-HSA was done by mixing 10 mg Se/L selenite to this HSA-stock solution and incubation for at least 14 days. Peak assignment for Se-HSA in CSF samples was done both, with Se-HSA and HSA monitoring selenium and UV peaks. Data files from selenium chromatograms were processed with the Clarity software for peak area integration. A typical Se-chromatogram is shown in the supplementary data (Supplemental Figure S1). We measured total serum selenium concentration in 1:3 diluted (Eluent A) CSF samples through inductively coupled plasma sector-field mass spectrometry (ICP-sf-MS). The experimental settings for ICP-sf-MS (ELEMENT II, Thermo Scientific, Bremen, Germany) were as follows: radio frequency power, 1260 W; plasma gas flow, 16 L Ar/min; auxiliary gas flow, 0.85 L Ar/min; nebulizer gas flow, 1.085 L Ar/min, daily optimized; dwell time, 300 ms; and ions monitored, ^77^Se and ^78^Se, using high-resolution mode. Five-point calibration curves from 0 to 5000 ng/L were linear with *r*^2^ for both Se isotopes being 0.9998.

### Analytical Quality Control for Selenium Speciation Analysis

We checked total selenium determination by analysis of a urine control material from Recipe, Munich, Germany (CSF control material was not available, but urine—like CSF—shows high salt but low protein concentration). We determined 22.1 ± 2.8 µg/L (target value 23 µg/L). Regularly, defined amounts of single selenium species standards were injected to the SAX-ICP-DRC-MS system, and peak selenium concentrations were quantified and related to the injected selenium amounts (= 100%) for recovery calculation. CSF samples were treated analogously. Recoveries for selenium standards ranged from 89 to 102%, whereas for CSF samples 97 ± 6% were found. Further, mass balances between the sum of quantified selenium species and total selenium determination were calculated, ranging between 91 and 103% for the entire CSF samples.

### Data Analysis

We computed descriptive statistics (median, 25th–75th percentiles, i.e., interquartile range) for all variables. We also estimated through linear regression analysis the relation between changes of selenium compounds and changes of ALS Functional Rating Scale (FRS), ALS Functional Rating Scale-Revised (Delta FS), and Forced Vital Capacity (FVC) overt time following tofersen treatment.

## Results

Table 1 summarizes the characteristics of the ten ALS patients carrying *SOD1* gene mutations in the study population, clinical features, and CSF concentrations of selenium species and overall selenium. The study participants included five males and five females, with spinal onset, classic or flail phenotypes, a median age of 58.6 years at baseline, and a median disease duration from first symptoms to diagnosis of 50 months. Among the various chemical forms of the element, organic Se-SELENOP was the compound with the highest concentration, followed by another organic form, co-eluting with Se-Cys_2_, and by inorganic tetravalent selenium, selenite. Baseline selenium compound concentrations according to exon location of the *SOD1* mutation and family history of the disease are listed in Supplemental Table S2, with little indication of major differences in selenium species according to these genetic factors.

**Table 1:** Median (50th) and interquartile range (IQR) of age (in years), neurofilaments (in pg/mL), and selenium concentrations (in µg/L) in cerebrospinal fluid in the study population according to sex (T0 = baseline; T6 = 6 months after treatment with tofersen)

	Sex-specific baseline values		T0		T6		Change (%)
	Males (*n* = 5)	Females (*n* = 5)	*N*	50th (IQR)	*N*	50th (IQR)	
Age	63.1 (54.5–65.1)	55.4 (50.7–61.9)					-
Neurofilaments	4483 (4435–12,058)	2035 (1788–3055)	10	4013 (2035–4843)	10	3280.5 (1172–4461)	− 18.3
Se-Total	2.04 (1.34–2.21)	1.48 (1.10–2.21)	10	1.49 (1.10–20.9)	10	2.21 (1.48–2.85)	48.3
Se-SELENOP	0.85 (0.80–1.05)	0.56 (0.53–1.02)	10	0.82 (0.53–1.05)	10	1.04 (0.83–1.36)	26.8
Se-Met	0.12 (0.12–0.14)	0.07 (0.00–0.08)	10	0.10 (0.07–0.12)	10	0.17 (0.07–0.23)	70.0
Se-Cys_2_	0.41 (0.17–0.43)	0.10 (0.01–0.12)	10	0.14 (0.10–0.41)	10	0.26 (0.06–0.40)	85.7
Se-GPX	0.07 (0.02–0.15)	0.04 (0.04–0.14)	10	0.06 (0.02–0.15)	10	0.08 (0.03–0.11)	33.3
Se-TXNRD	0.05 (0.004–0.09)	0.004 (0.004–0.02)	10	0.01 (0.01–0.07)	10	0.04 (0.01–0.10)	300.0
Se-(IV)	0.16 (0.10–0.16)	0.09 (0.07–0.13)	10	0.12 (0.09–0.16)	10	0.19 (0.15–0.27)	58.3
Se-(VI)	0.12 (0.07–0.15)	0.06 (0.05–0.06)	10	0.07 (0.06–0.15)	10	0.13 (0.07–0.23)	85.7
Se-HSA	0.03 (0.004–0.12)	0.004 (0.004–0.004)	10	0.004 (0.004–0.12)	10	0.04 (0.02–0.08)	900.0

Note: *Se-Cys*_*2*_, compound co-eluting with the selenocystine standard; *Se-GPX*, glutathione-peroxidase-bound selenium; *Se-HSA*, human serum albumin-bound selenium; *Se-Met*, selenomethionine-bound selenium; *Se-SELENOP*, selenoprotein P-bound selenium; *Se-TXNRD*, thioredoxin reductase-bound selenium; *Se(IV)*, selenite; *Se(VI)*, selenate

**Fig. 1:**
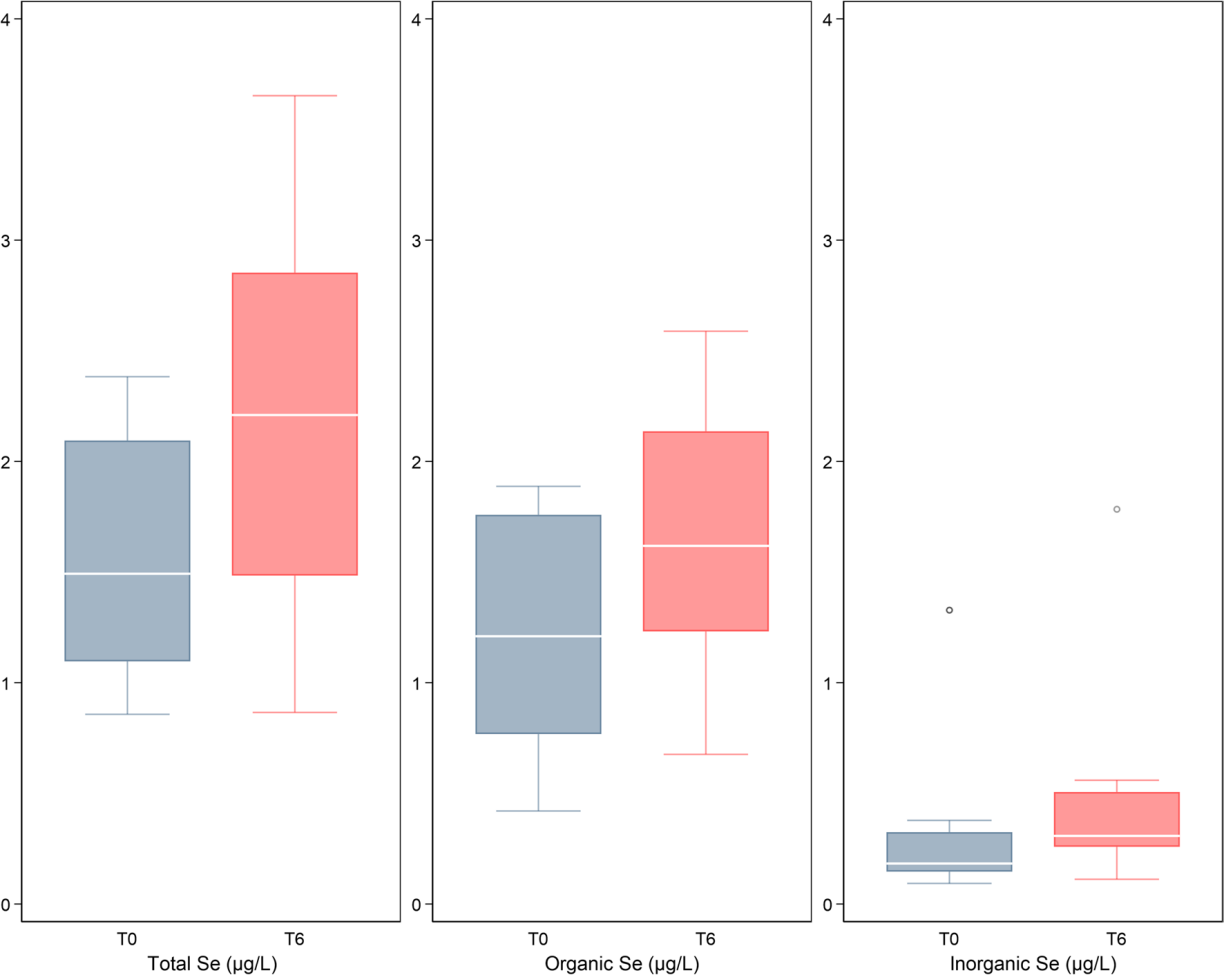
Boxplots of median concentrations in cerebrospinal fluid (in µg/L) of total selenium (Se) along with sum of organic and inorganic Se before and 6 months after treatment with tofersen. Notes: Se-Cys_2_, compound co-eluting with the selenocystine standard; Se-GPX, glutathione-peroxidase-bound selenium; Se-HSA, human serum albumin-bound selenium; Se-Met, selenomethionine-bound selenium; Se-SELENOP, selenoprotein P-bound selenium; Se-TXNRD, thioredoxin reductase-bound selenium; Se(IV), selenite; Se(VI), selenate

**Fig. 2:**
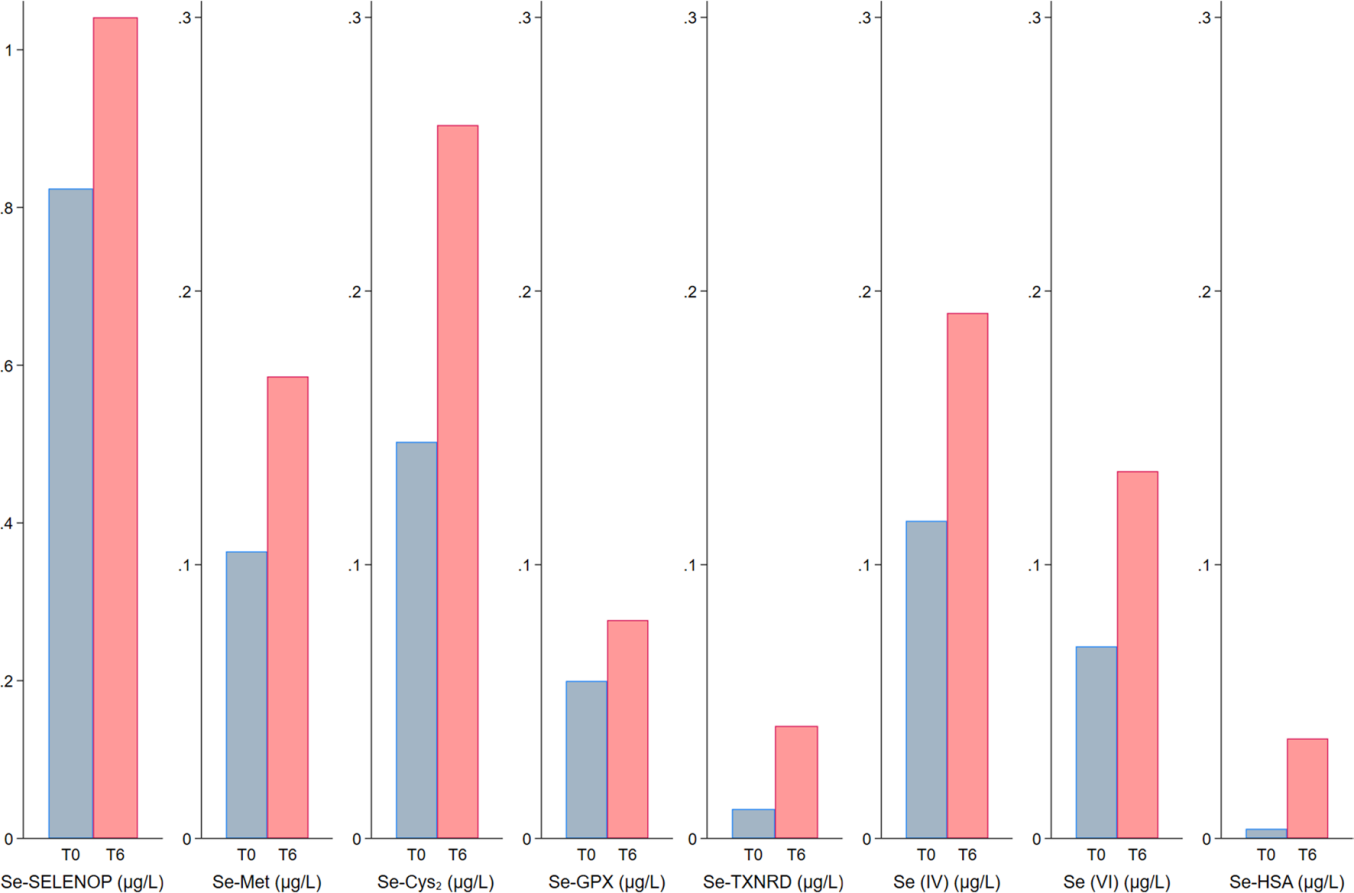
Boxplots of median organic and inorganic (Se) compound concentrations in cerebrospinal fluid (in µg/L) before and 6 months after tofersen treatment. Notes: Se-Cys_2_, compound co-eluting with the selenocystine standard; Se-GPX, glutathione-peroxidase-bound selenium; Se-HSA, human serum albumin-bound selenium; Se-Met, selenomethionine-bound selenium; Se-SELENOP, selenoprotein P-bound selenium; Se-TXNRD, thioredoxin reductase-bound selenium; Se(IV), selenite; Se(VI), selenate

Following tofersen treatment, there was a notable increase in CSF concentrations of various selenium chemical forms, encompassing overall selenium, the sum of organic, and the sum of inorganic chemical entities (Figs. 1 and 2). This increase was observed across almost all selenium species, except for the organic compound Se-GPX, whose concentrations remained substantially unchanged. The most substantial changes were observed for Se-TXNRD and Se-HSA, both exhibiting median concentrations approximately four times higher after the 6-month treatment period compared with baseline. A post-treatment increase in selenium species concentrations also clearly emerged for the Se-compound appearing at a retention time of Se-Cys_2_ and hexavalent inorganic selenium, selenate, whose median values were approximately twice as high compared with baseline concentrations. Conversely, the most abundant selenium species, Se-SELENOP, and another organic selenium form, Se-GPX, were those showing the lowest increases over time, slightly less, and more than 30%, respectively. Median CSF neurofilament concentration decreased by nearly 20% after tofersen administration, from 4013 to 3281 pg/mL, while corresponding mean concentrations decreased from 4947 to 3677 pg/mL (− 26%).

In linear regression analysis, changes in selenium compounds after tofersen treatment were generally negatively associated with changes in clinical parameters, particularly the ALS Functional Rating Scale and the Forced Vital Capacity, with some inconsistencies between the species and between the clinical endpoints (Supplemental Table S3 and Figures S2-S4).

## Discussion

In this study, we aimed at assessing if a 6-month administration of tofersen, a specific drug for the treatment of ALS associated with *SOD1* gene mutations, could modify the distribution of the metalloid selenium in the CNS, and more specifically in CSF. We pursued this objective in light of the key role of selenium, and particularly some chemical forms of this element in the CNS. These include its specific motor neuron toxicity as well as its pro-oxidant, antioxidant, and neurotoxic properties, also related to the different activities of selenocompounds on redox status and more generally in biological systems [20–22, 24, 36, 50–52]. Though in familial ALS gene mutations are known to be the key drivers of the disease, even in such cases, there is epidemiologic evidence that lifestyle and environmental risk factors are likely interacting with the genetic background [53–55].

Overall, we found that tofersen administration increased selenium levels in our study population, with a rather uneven pattern across the chemical forms of this metalloid. The most abundant selenium species, both at baseline and after drug administration, was as expected selenoprotein P, a selenium-transporter enzyme. Such an increase substantially drives the increase for overall selenium. Both beneficial and adverse properties have been attributed to selenoprotein P in the CNS and more generally in the body [36, 56–58]. Another relevant finding of the present study is a major increase in a selenium species of controversial chemical composition and function, Se-HSA, after tofersen administration [59]. In addition, selenium was also found to be bound to thioredoxin reductase, a cytosolic and mitochondrial antioxidant enzyme with a key role in redox reactions [60, 61] that has been shown to have an unexpectedly high CSF/serum ratio alongside Se-GPX, possibly indicative of either production in the brain or facilitated diffusion mechanism [62]. We also observed that tofersen induced a considerable increase in the CSF of three major neurotoxic selenium species, the organic form selenomethionine-bound selenium, and two inorganic selenium forms, selenite and selenate [29, 63–66]. These two species, in particular, have been associated with an excess ALS risk in epidemiologic studies with a case–control [28, 33] or cohort design [67]. Such an association is also supported by strong biological plausibility specifically referred to these species, whose neurotoxicity appears to be higher than the organic forms [30, 68–71]. In particular, in neuronal cells, inorganic selenium species are known to actively generate free radicals, alter the cytoskeleton by inducing microtubule defects, affect neurite length, and induce proteomic changes [25, 29, 32, 64, 72, 73]. Interestingly, selenium administration to human neuron cells also appears to interact with wild-type SOD1 expression by decreasing it and changing its localization from cytosol to mitochondria, in the perinuclear region [69]. Since no reference levels of CSF selenium species are available, we could not compare our results with such levels, though we generally found comparable results with other speciation studies in sporadic and familial ALS patients and in control subjects [28, 33].

In our study, there was a relevant decrease in the CSF concentrations of light chain neurofilaments following the drug treatment, confirming its biological activity. This decrease was less pronounced than that observed in the VALOR trial conducted in the USA [17] and within an Expanded Access Program in Germany [74, 75]. This may be due to the different matrix (CSF in our study as opposed to serum/plasma in the other studies), as well as some heterogeneity of study participants.

Our study has a few relevant limitations. First, the number of participants was not large enough to allow for a good precision in the effect estimates, as indicated by some statistical instability in the estimates. Secondly, we acknowledge that biochemical findings detected in the CSF cannot be easily correlated to changes occurring in the motor neurons. Therefore, the higher CSF contents of neurotoxic selenium species induced by tofersen treatment may reflect a decrease in such species within the motor neurons. This may be a reaction to lumbar puncture repetition, or a phenomenon related to disease progression itself, something that we could not entirely rule out due to the unavailability of a control population and the unfeasibility of CSF monitoring over time. However, we consider this scenario as very unlikely, given the large changes we detected in CSF selenium concentrations in a relatively short period of time, and more importantly, circulating selenium levels have been shown to inversely correlate with ALS progression, since serum selenium decreases with increasing disability according to a disease severity scale [76].

The changes we observed might also be accounted for as an effect of treatment-induced changes in the blood–brain barrier or in the redox status of the neuronal cells, in line with other recently reported effects [77, 78], thus inducing a compensatory, beneficial response against free radicals, including an upregulation of selenium-containing antioxidant enzymes. However, tofersen treatment also induced an increase in “non-physiological” selenium species devoid of antioxidant properties and potentially neurotoxic, such as inorganic (tetravalent and hexavalent) selenium and selenomethionine. The possibility that tofersen treatment could have enhanced selenium absorption into the brain, with beneficial or toxicological implications, or have altered its metabolism and excretion must also be considered. Finally, our results apply to carriers of *SOD1* gene mutations, but such mutations encompass a group of mutations with some heterogeneity [79], and it is possible that the effects of tofersen on CSF selenium levels preferentially occur in some of these different mutations of the same gene, suggesting the need of further research in larger series of patients. However, when we analyzed results according to specific characteristics of the *SOD1* mutation in study participants, we found little evidence of an effect of such genetic heterogeneity.

Selenium has already been acknowledged as a neurotoxic metalloid also involving motor function [80]. The possibility that it may be involved in ALS etiology was originally raised based on a cluster observed in a seleniferous area of South Dakota [27], followed by results from a natural experiment in Northern Italy [67]. Such observations have been further supported by a few case–control studies using CNS-based biomarkers [28, 31], including the only investigation so far to have been carried out in ALS-related gene mutation carriers [33]. In addition, biological plausibility for a role of selenium in ALS etiology, and particularly of its neurotoxic forms, has been provided by *in vitro* experiments [29, 30], along with the specific motor neuron toxicity of these selenium species in swine [70, 81, 82]. Overall, the results from the present study indicate that tofersen administration influences the CNS distribution of selenium species including the neurotoxic ones, a finding that could be related to disease etiology and progression.

## Supplementary Information

Below is the link to the electronic supplementary material.

Supplementary file1 (DOCX 346 KB)

## Data Availability

Anonymized data will be available upon reasonable request from the qualified investigators.

## References

[1] Goutman SA, Hardiman O, Al-Chalabi A, Chio A, Savelieff MG, Kiernan MC et al (2022) Emerging insights into the complex genetics and pathophysiology of amyotrophic lateral sclerosis. Lancet Neurol 21(5):465–479. 10.1016/S1474-4422(21)00414-2 35334234 10.1016/S1474-4422(21)00414-2 PMC9513754

[2] Martinelli I, Ghezzi A, Zucchi E, Gianferrari G, Ferri L, Moglia C et al (2023) Predictors for progression in amyotrophic lateral sclerosis associated to SOD1 mutation: insight from two population-based registries. J Neurol 270(12):6081–6092. 10.1007/s00415-023-11963-0 37668704 10.1007/s00415-023-11963-0

[3] Rosen DR (1993) Mutations in Cu/Zn superoxide dismutase gene are associated with familial amyotrophic lateral sclerosis. Nature 364(6435):362. 10.1038/364362c0 8332197 10.1038/364362c0

[4] Rakhit R, Chakrabartty A (2006) Structure, folding, and misfolding of Cu, Zn superoxide dismutase in amyotrophic lateral sclerosis. Biochim Biophys Acta 1762(11–12):1025–1037. 10.1016/j.bbadis.2006.05.004 16814528 10.1016/j.bbadis.2006.05.004

[5] Park JH, Nordstrom U, Tsiakas K, Keskin I, Elpers C, Mannil M et al (2023) The motor system is exceptionally vulnerable to absence of the ubiquitously expressed superoxide dismutase-1. Brain Commun 5(1):fcad017. 10.1093/braincomms/fcad017 36793789 10.1093/braincomms/fcad017 PMC9924500

[6] Vinceti M, Bottecchi I, Fan A, Finkelstein Y, Mandrioli J (2012) Are environmental exposures to selenium, heavy metals, and pesticides risk factors for amyotrophic lateral sclerosis? Rev Environ Health 27(1):19–41. 10.1515/reveh-2012-0002 22755265 10.1515/reveh-2012-0002

[7] Jin J, Zhong XB (2023) ASO drug Qalsody (tofersen) targets amyotrophic lateral sclerosis. Trends Pharmacol Sci 44(12):1043–1044. 10.1016/j.tips.2023.08.008 37709589 10.1016/j.tips.2023.08.008 PMC10841252

[8] Saini A, Chawla PA (2024) Breaking barriers with tofersen: enhancing therapeutic opportunities in amyotrophic lateral sclerosis. Eur J Neurol 31(2):e16140. 10.1111/ene.16140 10.1111/ene.16140 PMC11235929 37975798

[9] van Roon-Mom W, Ferguson C, Aartsma-Rus A (2023) From failure to meet the clinical endpoint to U.S. Food and Drug Administration Approval: 15th Antisense oligonucleotide therapy approved qalsody (tofersen) for treatment of SOD1 mutated amyotrophic lateral sclerosis. Nucleic Acid Ther. 33(4):234–7. 10.1089/nat.2023.0027 37581487 10.1089/nat.2023.0027

[10] Sabatelli M, Cerri F, Zuccarino R, Patanella AK, Bernardo D, Bisogni G et al (2024) Long-term treatment of SOD1 ALS with tofersen: a multicentre experience in 17 patients. J Neurol. 10.1007/s00415-024-12437-7 38829431 10.1007/s00415-024-12437-7

[11] Boros BD, Schoch KM, Kreple CJ, Miller TM (2022) Antisense oligonucleotides for the study and treatment of ALS. Neurotherapeutics 19(4):1145–1158. 10.1007/s13311-022-01247-2 35653060 10.1007/s13311-022-01247-2 PMC9587169

[12] Meijboom KE, Brown RH (2022) Approaches to gene modulation therapy for ALS. Neurotherapeutics 19(4):1159–1179. 10.1007/s13311-022-01285-w 36068427 10.1007/s13311-022-01285-w PMC9587165

[13] Kim G, Gautier O, Tassoni-Tsuchida E, Ma XR, Gitler AD (2020) ALS genetics: gains, losses, and implications for future therapies. Neuron 108(5):822–842. 10.1016/j.neuron.2020.08.022 32931756 10.1016/j.neuron.2020.08.022 PMC7736125

[14] Martinelli I, Zucchi E, Simonini C, Gianferrari G, Zamboni G, Pinti M et al (2023) The landscape of cognitive impairment in superoxide dismutase 1-amyotrophic lateral sclerosis. Neural Regen Res 18(7):1427–1433. 10.4103/1673-5374.361535 36571338 10.4103/1673-5374.361535 PMC10075107

[15] Miller T, Cudkowicz M, Shaw PJ, Andersen PM, Atassi N, Bucelli RC et al (2020) Phase 1–2 trial of antisense oligonucleotide tofersen for SOD1 ALS. N Engl J Med 383(2):109–119. 10.1056/NEJMoa2003715 32640130 10.1056/NEJMoa2003715

[16] Benatar M, Wuu J, Andersen PM, Bucelli RC, Andrews JA, Otto M et al (2022) Design of a randomized, placebo-controlled, phase 3 trial of tofersen initiated in clinically presymptomatic SOD1 variant carriers: the ATLAS study. Neurotherapeutics 19(4):1248–1258. 10.1007/s13311-022-01237-4 35585374 10.1007/s13311-022-01237-4 PMC9587202

[17] Miller TM, Cudkowicz ME, Genge A, Shaw PJ, Sobue G, Bucelli RC et al (2022) Trial of antisense oligonucleotide tofersen for SOD1 ALS. N Engl J Med 387(12):1099–1110. 10.1056/NEJMoa2204705 36129998 10.1056/NEJMoa2204705

[18] Saini A, Chawla PA (2024) Breaking barriers with tofersen: enhancing therapeutic opportunities in amyotrophic lateral sclerosis. Eur J Neurol 31(2):e16140. 10.1111/ene.16140 37975798 10.1111/ene.16140 PMC11235929

[19] Labunskyy VM, Hatfield DL, Gladyshev VN (2014) Selenoproteins: molecular pathways and physiological roles. Physiol Rev 94(3):739–777. 10.1152/physrev.00039.2013 24987004 10.1152/physrev.00039.2013 PMC4101630

[20] Jablonska E, Vinceti M (2015) Selenium and human health: witnessing a Copernican revolution? J Environ Sci Health Part C Environ Carcinog Ecotoxicol Rev 33(3):328–368. 10.1080/10590501.2015.1055163 10.1080/10590501.2015.1055163 26074278

[21] Saito Y (2020) Selenoprotein P as an in vivo redox regulator: disorders related to its deficiency and excess. J Clin Biochem Nutr 66(1):1–7. 10.3164/jcbn.19-31 32001950 10.3164/jcbn.19-31 PMC6983434

[22] Vinceti M, Filippini T, Jablonska E, Saito Y, Wise LA (2022) Safety of selenium exposure and limitations of selenoprotein maximization: molecular and epidemiologic perspectives. Environ Res 211:113092. 10.1016/j.envres.2022.113092 35259406 10.1016/j.envres.2022.113092

[23] Hatfield DL, Tsuji PA, Carlson BA, Gladyshev VN (2014) Selenium and selenocysteine: roles in cancer, health, and development. Trends Biochem Sci 39(3):112–120. 10.1016/j.tibs.2013.12.007 24485058 10.1016/j.tibs.2013.12.007 PMC3943681

[24] Dauplais M, Romero S, Lazard M (2024) Exposure to selenomethionine and selenocystine induces redox-mediated ER stress in normal breast epithelial MCF-10A cells. Biol Trace Elem Res. 10.1007/s12011-024-04244-y 38777874 10.1007/s12011-024-04244-y

[25] Lin Y, Hu L, Li X, Ma J, Li Q, Yuan X et al (2024) The beneficial and toxic effects of selenium on zebrafish. A systematic review of the literature. Toxicol Res (Camb) 13(2):tfae062. 10.1093/toxres/tfae062 38645626 10.1093/toxres/tfae062 PMC11031411

[26] Hafeman DG, Sunde RA, Hoekstra WG (1974) Effect of dietary selenium on erythrocyte and liver glutathione peroxidase in the rat. J Nutr 104:580–587. 10.1093/jn/104.5.580 4823943 10.1093/jn/104.5.580

[27] Kilness AW, Hichberg FH (1977) Amyotrophic lateral sclerosis in a high selenium environment. JAMA 237(26):2843–2844. 10.1001/jama.1977.03270530051023 577250

[28] Vinceti M, Solovyev N, Mandrioli J, Crespi CM, Bonvicini F, Arcolin E et al (2013) Cerebrospinal fluid of newly diagnosed amyotrophic lateral sclerosis patients exhibits abnormal levels of selenium species including elevated selenite. Neurotoxicology 38:25–32. 10.1016/j.neuro.2013.05.016 23732511 10.1016/j.neuro.2013.05.016 PMC3770807

[29] Vinceti M, Mandrioli J, Borella P, Michalke B, Tsatsakis A, Finkelstein Y (2014) Selenium neurotoxicity in humans: bridging laboratory and epidemiologic studies. Toxicol Lett 230(2):295–303. 10.1016/j.toxlet.2013.11.016 24269718 10.1016/j.toxlet.2013.11.016

[30] Naderi M, Puar P, Zonouzi-Marand M, Chivers DP, Niyogi S, Kwong RWM (2021) A comprehensive review on the neuropathophysiology of selenium. Sci Total Environ 767:144329. 10.1016/j.scitotenv.2020.144329 33445002 10.1016/j.scitotenv.2020.144329

[31] Kamalian A, Foroughmand I, Koski L, Darvish M, Saghazadeh A, Kamalian A et al (2023) Metal concentrations in cerebrospinal fluid, blood, serum, plasma, hair, and nails in amyotrophic lateral sclerosis: a systematic review and meta-analysis. J Trace Elem Med Biol 78:127165. 10.1016/j.jtemb.2023.127165 37018859 10.1016/j.jtemb.2023.127165

[32] Filippini T, Michalke B, Mandrioli J, Tsatsakis A, Weuve J, Vinceti M (2018) Selenium neurotoxicity and amyotrophic lateral sclerosis: an epidemiologic perspective. In: Michalke B (ed) Selenium molecular and integrative toxicology. Cham, Springer, pp 231–248. 10.1007/978-3-319-95390-8_12

[33] Mandrioli J, Michalke B, Solovyev N, Grill P, Violi F, Lunetta C et al (2017) Elevated levels of selenium species in cerebrospinal fluid of amyotrophic lateral sclerosis patients with disease-associated gene mutations. Neurodegener Dis 17(4–5):171–180. 10.1159/000460253 28478440 10.1159/000460253

[34] Adani G, Filippini T, Michalke B, Vinceti M (2020) Selenium and other trace elements in the etiology of Parkinson’s disease: a systematic review and meta-analysis of case-control studies. Neuroepidemiology 54(1):1–23. 10.1159/000502357 31454800 10.1159/000502357

[35] Vinceti M, Balboni E, Filippini T, Wise LA, Nocetti L, Eichmuller M et al (2022) Selenium species in cerebrospinal fluid and hippocampal volume among individuals with mild cognitive impairment. Environ Health Perspect 130(11):117701. 10.1289/EHP11445 36331818 10.1289/EHP11445 PMC9635506

[36] Vinceti M, Urbano T, Chiari A, Filippini T, Wise LA, Tondelli M et al (2023) Selenoprotein P concentrations and risk of progression from mild cognitive impairment to dementia. Sci Rep 13(1):8792. 10.1038/s41598-023-36084-6 37258587 10.1038/s41598-023-36084-6 PMC10232449

[37] Wang J, Huang Y, Bei C, Yang H, Lin Z, Xu L (2024) Causal associations of antioxidants with Alzheimer’s disease and cognitive function: a Mendelian randomisation study. J Epidemiol Community Health 78(7):424–430. 10.1136/jech-2023-221184 38589220 10.1136/jech-2023-221184

[38] Vinceti M, Grill P, Malagoli C, Filippini T, Storani S, Malavolti M et al (2015) Selenium speciation in human serum and its implications for epidemiologic research: a cross-sectional study. J Trace Elem Med Biol 31:1–10. 10.1016/j.jtemb.2015.02.001 26004885 10.1016/j.jtemb.2015.02.001

[39] Urbano T, Filippini T, Wise LA, Sucato S, Polledri E, Malavolti M et al (2023) Selenium exposure and urinary 8-oxo-7,8-dihydro-2’-deoxyguanosine: major effects of chemical species and sex. Sci Total Environ 870:161584. 10.1016/j.scitotenv.2023.161584 36702271 10.1016/j.scitotenv.2023.161584

[40] Urbano T, Filippini T, Malavolti M, Fustinoni S, Michalke B, Wise LA et al (2024) Adherence to the Mediterranean-DASH Intervention for Neurodegenerative Delay (MIND) diet and exposure to selenium species: a cross-sectional study. Nutr Res 122:44–54. 10.1016/j.nutres.2023.12.002 38150803 10.1016/j.nutres.2023.12.002

[41] Zhang C, Zeng Q, Liu X, He Q, Zhang J, Zhao S et al (2024) Association of blood selenium levels with diabetes and heart failure in American general adults: a cross-sectional study of NHANES 2011–2020 pre. Biol Trace Elem Res 202(8):3413–3424. 10.1007/s12011-023-03933-4 37996718 10.1007/s12011-023-03933-4 PMC11144148

[42] Vinceti M, Filippini T, Wise LA, Rothman KJ (2021) A systematic review and dose-response meta-analysis of exposure to environmental selenium and the risk of type 2 diabetes in nonexperimental studies. Environ Res 197:111210. 10.1016/j.envres.2021.111210 33895112 10.1016/j.envres.2021.111210

[43] EFSA Panel on Nutrition Novel Foods Food Allergens, Turck D, Bohn T, Castenmiller J, de Henauw S, Hirsch-Ernst KI et al (2023) Scientific opinion on the tolerable upper intake level for selenium. EFSA J 21(1):e07704. 10.2903/j.efsa.2023.7704 10.2903/j.efsa.2023.7704 PMC9854220 36698500

[44] Simonini C, Zucchi E, Bedin R, Martinelli I, Gianferrari G, Fini N et al (2021) CSF heavy neurofilament may discriminate and predict motor neuron diseases with upper motor neuron involvement. Biomedicines 9(11):1623. 10.3390/biomedicines9111623 34829852 10.3390/biomedicines9111623 PMC8615649

[45] Martinelli I, Zucchi E, Simonini C, Gianferrari G, Bedin R, Biral C et al (2024) SerpinA1 levels in amyotrophic lateral sclerosis patients: an exploratory study. Eur J Neurol 31(1):e16054. 10.1111/ene.16054 37679868 10.1111/ene.16054 PMC11235621

[46] Shigeta K, Sato K, Furuta N (2007) Determination of selenoprotein P in submicrolitre samples of human plasma using micro-affinity chromatography coupled with low flow ICP-MS. J Anal At Spectrom 22(8):911–916. 10.1039/B701206C

[47] Jitaru P, Prete M, Cozzi G, Turetta C, Cairns W, Seraglia R et al (2008) Speciation analysis of selenoproteins in human serum by solid-phase extraction and affinity HPLC hyphenated to ICP-quadrupole MS. J Anal At Spectrom 23(3):402–406. 10.1039/B712693J

[48] Solovyev N, Berthele A, Michalke B (2013) Selenium speciation in paired serum and cerebrospinal fluid samples. Anal Bioanal Chem 405(6):1875–1884. 10.1007/s00216-012-6294-y 22868477 10.1007/s00216-012-6294-y

[49] Ballihaut G, Kilpatrick LE, Kilpatrick EL, Davis WC (2012) Multiple forms of selenoprotein P in a candidate human plasma standard reference material. Metallomics 4(6):533–538. 10.1039/C2MT20059G 22552441 10.1039/c2mt20059g

[50] Labunskyy VM, Lee BC, Handy DE, Loscalzo J, Hatfield DL, Gladyshev VN (2011) Both maximal expression of selenoproteins and selenoprotein deficiency can promote development of type 2 diabetes-like phenotype in mice. Antioxid Redox Signal 14(12):2327–2336. 10.1089/ars.2010.3526 21194350 10.1089/ars.2010.3526 PMC3096499

[51] Tsuji PA, Carlson BA, Yoo MH, Naranjo-Suarez S, Xu XM, He Y et al (2015) The 15kDa selenoprotein and thioredoxin reductase 1 promote colon cancer by different pathways. PLoS One 10(4):e0124487. 10.1371/journal.pone.0124487 25886253 10.1371/journal.pone.0124487 PMC4401539

[52] Canter JA, Ernst SE, Peters KM, Carlson BA, Thielman NRJ, Grysczyk L et al (2021) Selenium and the 15kDa selenoprotein impact colorectal tumorigenesis by modulating intestinal barrier integrity. Int J Mol Sci 22(19):10651. 10.3390/ijms221910651 34638991 10.3390/ijms221910651 PMC8508755

[53] Graham AJ, Macdonald AM, Hawkes CH (1997) British motor neuron disease twin study. J Neurol Neurosurg Psychiatry 62(6):562–569. 10.1136/jnnp.62.6.562 9219739 10.1136/jnnp.62.6.562 PMC1074137

[54] Xi Z, Yunusova Y, van Blitterswijk M, Dib S, Ghani M, Moreno D et al (2014) Identical twins with the C9orf72 repeat expansion are discordant for ALS. Neurology 83(16):1476–1478. 10.1212/WNL.0000000000000886 25209579 10.1212/WNL.0000000000000886 PMC4206161

[55] Goutman SA, Savelieff MG, Jang DG, Hur J, Feldman EL (2023) The amyotrophic lateral sclerosis exposome: recent advances and future directions. Nat Rev Neurol 19(10):617–634. 10.1038/s41582-023-00867-2 37709948 10.1038/s41582-023-00867-2 PMC11027963

[56] Schweizer U, Fabiano M (2022) Selenoproteins in brain development and function. Free Radic Biol Med 190:105–115. 10.1016/j.freeradbiomed.2022.07.022 35961466 10.1016/j.freeradbiomed.2022.07.022

[57] Solovyev N, Drobyshev E, Blume B, Michalke B (2021) Selenium at the neural barriers: a review. Front Neurosci 15:630016. 10.3389/fnins.2021.630016 33613188 10.3389/fnins.2021.630016 PMC7892976

[58] Urbano T, Vinceti M, Mandrioli J, Chiari A, Filippini T, Bedin R et al (2022) Selenoprotein P concentrations in the cerebrospinal fluid and serum of individuals affected by amyotrophic lateral sclerosis, mild cognitive impairment and Alzheimer’s dementia. Int J Mol Sci 23(17):9865. 10.3390/ijms23179865 36077261 10.3390/ijms23179865 PMC9456314

[59] Filippini T, Urbano T, Grill P, Malagoli C, Ferrari A, Marchesi C et al (2023) Human serum albumin-bound selenium (Se-HSA) in serum and its correlation with other selenium species. J Trace Elem Med Biol 79:127266. 10.1016/j.jtemb.2023.127266 37499550 10.1016/j.jtemb.2023.127266

[60] Bjorklund G, Zou L, Wang J, Chasapis CT, Peana M (2021) Thioredoxin reductase as a pharmacological target. Pharmacol Res 174:105854. 10.1016/j.phrs.2021.105854 34455077 10.1016/j.phrs.2021.105854

[61] Gopalakrishna R, Gundimeda U, Zhou S, Bui H, Holmgren A (2018) Redox regulation of protein kinase C by selenometabolites and selenoprotein thioredoxin reductase limits cancer prevention by selenium. Free Radic Biol Med 127:55–61. 10.1016/j.freeradbiomed.2018.05.062 29775743 10.1016/j.freeradbiomed.2018.05.062

[62] Michalke B, Willkommen D, Drobyshev E, Solovyev N (2018) The importance of speciation analysis in neurodegeneration research. TrAC, Trends Anal Chem 104:160–170. 10.1016/j.trac.2017.08.008

[63] Naderi M, Salahinejad A, Ferrari MCO, Niyogi S, Chivers DP (2018) Dopaminergic dysregulation and impaired associative learning behavior in zebrafish during chronic dietary exposure to selenium. Environ Pollut 237:174–185. 10.1016/j.envpol.2018.02.033 29482023 10.1016/j.envpol.2018.02.033

[64] Maraldi T, Beretti F, Anselmi L, Franchin C, Arrigoni G, Braglia L et al (2019) Influence of selenium on the emergence of neuro tubule defects in a neuron-like cell line and its implications for amyotrophic lateral sclerosis. Neurotoxicology 75:209–220. 10.1016/j.neuro.2019.09.015 31585128 10.1016/j.neuro.2019.09.015

[65] Lee JW, Deng DF, Lee J, Kim K, Jung HJ, Choe Y et al (2020) The adverse effects of selenomethionine on skeletal muscle, liver, and brain in the steelhead trout (*Oncorhynchus mykiss*). Environ Toxicol Pharmacol 80:103451. 10.1016/j.etap.2020.103451 10.1016/j.etap.2020.103451 32599160

[66] Vinceti M, Chiari A, Eichmuller M, Rothman KJ, Filippini T, Malagoli C et al (2017) A selenium species in cerebrospinal fluid predicts conversion to Alzheimer’s dementia in persons with mild cognitive impairment. Alzheimers Res Ther 9(1):100. 10.1186/s13195-017-0323-1 29258624 10.1186/s13195-017-0323-1 PMC5735937

[67] Vinceti M, Filippini T, Malagoli C, Violi F, Mandrioli J, Consonni D et al (2019) Amyotrophic lateral sclerosis incidence following exposure to inorganic selenium in drinking water: a long-term follow-up. Environ Res 179(Pt A):108742. 10.1016/j.envres.2019.108742 31629180 10.1016/j.envres.2019.108742

[68] Ammar EM, Couri D (1981) Acute toxicity of sodium selenite and selenomethionine in mice after ICV or IV administration. Neurotoxicology 2(2):383–386 7198759

[69] Maraldi T, Riccio M, Zambonin L, Vinceti M, De Pol A, Hakim G (2011) Low levels of selenium compounds are selectively toxic for a human neuron cell line through ROS/RNS increase and apoptotic process activation. Neurotoxicology 32(2):180–187. 10.1016/j.neuro.2010.10.008 21215776 10.1016/j.neuro.2010.10.008

[70] Panter KE, Hartley WJ, James LF, Mayland HF, Stegelmeier BL, Kechele PO (1996) Comparative toxicity of selenium from seleno-DL-methionine, sodium selenate, and *Astragalus bisulcatus* in pigs. Fund Appl Toxicol 32(2):217–223 8921324

[71] Rohn I, Marschall TA, Kroepfl N, Jensen KB, Aschner M, Tuck S et al (2018) Selenium species-dependent toxicity, bioavailability and metabolic transformations in *Caenorhabditis elegans.* Metallomics 10(6):818–827. 10.1039/c8mt00066b 10.1039/c8mt00066b PMC6013411 29770420

[72] Ullah H, Liu G, Yousaf B, Ali MU, Irshad S, Abbas Q et al (2019) A comprehensive review on environmental transformation of selenium: recent advances and research perspectives. Environ Geochem Health 41(2):1003–1035. 10.1007/s10653-018-0195-8 30267320 10.1007/s10653-018-0195-8

[73] Kim JH, Kang JC (2015) Oxidative stress, neurotoxicity, and non-specific immune responses in juvenile red sea bream, *Pagrus major*, exposed to different waterborne selenium concentrations. Chemosphere 135:46–52. 10.1016/j.chemosphere.2015.03.062 10.1016/j.chemosphere.2015.03.062 25898389

[74] Meyer T, Schumann P, Weydt P, Petri S, Koc Y, Spittel S et al (2023) Neurofilament light-chain response during therapy with antisense oligonucleotide tofersen in SOD1-related ALS: treatment experience in clinical practice. Muscle Nerve 67(6):515–521. 10.1002/mus.27818 10.1002/mus.27818 36928619

[75] Wiesenfarth M, Dorst J, Brenner D, Elmas Z, Parlak O, Uzelac Z et al (2024) Effects of tofersen treatment in patients with SOD1-ALS in a “real-world” setting - a 12-month multicenter cohort study from the German early access program. EClinicalMedicine 69:102495. 10.1016/j.eclinm.2024.102495 38384337 10.1016/j.eclinm.2024.102495 PMC10878861

[76] Vinceti M, Guidetti D, Bergomi M, Caselgrandi E, Vivoli R, Olmi M et al (1997) Lead, cadmium, and selenium in the blood of patients with sporadic amyotrophic lateral sclerosis. Ital J Neurol Sci 18(2):87–92. 10.1007/BF01999568 9239528 10.1007/BF01999568

[77] Sparasci D, Castelli C, Staedler C, Gobbi C, Ripellino P (2023) Inclusions in macrophages of the cerebrospinal fluid during treatment with Tofersen. Muscle Nerve 67(2):E3–E5. 10.1002/mus.27763 36477882 10.1002/mus.27763

[78] Reilich P, Schoberl F, Hiebeler M, Tonon M, Ludolph AC, Senel M (2024) Myelitis as a side effect of tofersen therapy in SOD1-associated ALS. J Neurol 271(4):2114–2118. 10.1007/s00415-023-12130-1 38066205 10.1007/s00415-023-12130-1 PMC10973064

[79] Martinelli I, Mandrioli J, Ghezzi A, Zucchi E, Gianferrari G, Simonini C et al (2025) Multifaceted superoxide dismutase 1 expression in amyotrophic lateral sclerosis patients: a rare occurrence? Neural Regen Res 20(1):130–138. 10.4103/NRR.NRR-D-23-01904 38767482 10.4103/NRR.NRR-D-23-01904 PMC11246149

[80] Yang GQ, Wang SZ, Zhou RH, Sun SZ (1983) Endemic selenium intoxication of humans in China. Am J Clin Nutr 37(5):872–881. 10.1093/ajcn/37.5.872 6846228 10.1093/ajcn/37.5.872

[81] Raber M, Sydler T, Wolfisberg U, Geyer H, Burgi E (2010) Feed-related selenium poisoning in swine. Schweiz Arch Tierheilkd 152(5):245–252. 10.1024/0036-7281/a000056 20464684 10.1024/0036-7281/a000056

[82] Casteignau A, Fontan A, Morillo A, Oliveros JA, Segales J (2006) Clinical, pathological and toxicological findings of a iatrogenic selenium toxicosis case in feeder pigs. J Vet Med A Physiol Pathol Clin Med 53(6):323–326. 10.1111/j.1439-0442.2006.00830.x 10.1111/j.1439-0442.2006.00830.x 16901278

