# Supplementary material for: Changes in Cerebrospinal Fluid Concentrations of Selenium Species Induced by Tofersen Administration in Subjects with Amyotrophic Lateral Sclerosis Carrying SOD1 Gene Mutations
Source: Biol Trace Elem Res. 2024 Jul 17;203(4):2355–64. doi: 10.1007/s12011-024-04311-4 (PMC11920394; doi:10.1007/s12011-024-04311-4)
Supplement: Supplementary file 1 — Supplementary file1 (DOCX 346 KB) [file 12011_2024_4311_MOESM1_ESM.docx]

**Table S1.** Characteristics of the SOD1 mutations in the patients included in this study who were treated with tofersen.

| Patient ID | Sex | Diagnostic latency, months | Age at onset, years | Age at baseline, years | SOD  mutation | Exon  location | Family history |
| --- | --- | --- | --- | --- | --- | --- | --- |
| 1 | M | 7.50 | 53.48 | 54.56 | E134del | 5 | no |
| 2 | M | 5.59 | 27.03 | 27.66 | A5V | 1 | yes |
| 3 | M | 24.59 | 61.66 | 65.10 | E134del | 5 | yes |
| 4 | F | 44.65 | 45.35 | 61.98 | A96T | 4 | yes |
| 5 | M | 29.59 | 61.74 | 68.29 | G11E | 1 | no |
| 6 | F | 18.08 | 59.27 | 62.18 | D91A | 4 | no |
| 7 | F | 67.33 | 40.70 | 50.69 | I150T | 5 | no |
| 8 | F | 26.93 | 40.53 | 44.61 | I150T | 5 | no |
| 9 | M | 42.05 | 56.36 | 63.12 | L145F | 5 | yes |
| 10 | F | 36.20 | 52.06 | 55.39 | E41G | 2 | yes |

**Table S2.** Median and interquartile range concentrations (µg/L) of selenium compounds at baseline according to location of the SOD mutation and family history of the disease.

|  | Exon location of SOD mutation | | Family history | |
| --- | --- | --- | --- | --- |
|  | **Exon 5 (N=5)** | **Other exon**  **(1, 2, 4) (N=5)** | **Yes (N=5)** | **No (N=5)** |
| Se-Total | 1.34 (1.10-1.48) | 2.04 (1.50-2.09) | 1.50 (1.34-2.04) | 1.48 (1.10-2.09) |
| Se-SELENOP | 0.85 (0.56-1.05) | 0.80 (0.53-1.02) | 1.02 (0.85-1.05) | 0.56 (0.36-0.80) |
| Se-Met | 0.12 (0.08-0.12) | 0.09 (0.07-0.12) | 0.12 (0.09-0.12) | 0.08 (0.00-0.12) |
| Se-Cys_2_ | 0.15 (0.10-0.17) | 0.14 (0.12-0.41) | 0.17 (0.14-0.41) | 0.10 (0.01-0.15) |
| Se-GPX | 0.04 (0.02-0.15) | 0.07 (0.04-0.14) | 0.04 (0.02-0.07) | 0.14 (0.04-0.16) |
| Se-TXNRD | 0.00 (0.00-0.02) | 0.05 (0.00-0.07) | 0.00 (0.00-0.05) | 0.02 (0.00-0.07) |
| Se(IV) | 0.10 (0.09-0.10) | 0.16 (0.13-0.17) | 0.13 (0.10-0.16) | 0.10 (0.09-0.17) |
| Se(VI) | 0.07 (0.06-0.07) | 0.12 (0.06-0.15) | 0.07 (0.06-0.12) | 0.07 (0.06-0.15) |
| Se-HSA | 0.00 (0.00-0.03) | 0.00 (0.00-0.12) | 0.00 (0.00-0.03) | 0.00 (0.00-0.12) |

Notes: Se-Cys_2_, compound co-eluting with the selenocystine standard; Se-GPX, glutathione-peroxidase-bound selenium; Se-HSA, human serum albumin-bound selenium; Se-Met, selenomethionine-bound selenium; Se-SELENOP, selenoprotein P-bound selenium; Se-TXNRD, thioredoxin reductase-bound selenium; Se(IV), selenite; Se(VI), selenate

**Table S3.** Regression analyses between changes in selenium compounds and Functional Rating Scale (FRS), rate of decline over time in the ALS Functional Rating Scale-Revised (Delta FS), and Forced Vital Capacity (FVC) of tofersen treated patients.

|  | β [95 % confidence interval] | | |
| --- | --- | --- | --- |
|  | **Δ FRS** | **Δ Delta FS** | **Δ FVC** |
| Δ Se-Total | -1.03  [-2.76, 0.70] | 0.03  [-0.16, 0.22] | -1.45  [-15.68, 12.78] |
| Δ Se-SELENOP | -1.26  [-4.65, 2.14] | 0.07  [-0.27, 0.42] | -4.66  [-34.48, 25.15] |
| Δ Se-Met | 6.02  [-8.88, 20.92] | -1.24  [-2.32, -0.15] | -44.61  [-54.15, -35.08] |
| Δ Se-Cys_2_ | -5.51  [-10.50, -0.52] | 0.14  [-0.53, 0.82] | 27.74  [-18.63, 74.12] |
| Δ Se-GPX | -8.56  [-20.76, 3.64] | -0.02  [-1.43, 1.39] | -7.48  [-84.67, 69.71] |
| Δ Se-TXNRD | -7.63  [-39.46, 24.20] | 0.82  [-2.28, 3.92] | -8.06  [-173.93, 157.82] |
| Δ Se (IV) | -7.25  [-26.94, 12.45] | 0.90  [-0.97, 2.76] | 2.61  [-105.23, 110.45] |
| Δ Se (VI) | -1.73  [-22.75, 19.29] | 0.47  [-1.55, 2.49] | -12.12  [-117.30, 93.07] |
| Δ Se-HSA | -23.79  [-52.67, 5.09] | -0.59  [-4.06, 2.88] | 57.01  [-96.96, 210.98] |

Notes: Δ: t6 – t0; Se-Cys_2_, compound co-eluting with the selenocystine standard; Se-GPX, glutathione-peroxidase-bound selenium; Se-HSA, human serum albumin-bound selenium; Se-Met, selenomethionine-bound selenium; Se-SELENOP, selenoprotein P-bound selenium; Se-TXNRD, thioredoxin reductase-bound selenium; Se(IV), selenite; Se(VI), selenate.

**Figure S1.** Chromatogram of a cerebrospinal fluid sample. Selenium species assignment was performed according to retention time matching with respective standard compounds (c.f. 2.2. analytical determinations).

Notes: Se-Cys_2_, compound co-eluting with the selenocystine standard; Se-GPX, glutathione-peroxidase-bound selenium; Se-HSA, human serum albumin-bound selenium; Se-Met, selenomethionine-bound selenium; Se-SELENOP, selenoprotein P-bound selenium; Se-TXNRD, thioredoxin reductase-bound selenium; Se(IV), selenite; Se(VI), selenate.

**Figure S2.** Regression analyses between changes in selenium compounds and Functional Rating Scale (FRS) of tofersen treated patients.

Notes: Se-Cys_2_, compound co-eluting with the selenocystine standard; Se-GPX, glutathione-peroxidase-bound selenium; Se-HSA, human serum albumin-bound selenium; Se-Met, selenomethionine-bound selenium; Se-SELENOP, selenoprotein P-bound selenium; Se-TXNRD, thioredoxin reductase-bound selenium; Se(IV), selenite; Se(VI), selenate.

**Figure S3.** Regression analyses between changes in selenium compounds and rate of decline over time in the ALS Functional Rating Scale-Revised (Delta FS) of tofersen treated patients.

Notes: Se-Cys_2_, compound co-eluting with the selenocystine standard; Se-GPX, glutathione-peroxidase-bound selenium; Se-HSA, human serum albumin-bound selenium; Se-Met, selenomethionine-bound selenium; Se-SELENOP, selenoprotein P-bound selenium; Se-TXNRD, thioredoxin reductase-bound selenium; Se(IV), selenite; Se(VI), selenate.

**Figure S4.** Regression analyses between changes in selenium compounds and Forced Vital Capacity (FVC) of tofersen treated patients.

Notes: Se-Cys_2_, compound co-eluting with the selenocystine standard; Se-GPX, glutathione-peroxidase-bound selenium; Se-HSA, human serum albumin-bound selenium; Se-Met, selenomethionine-bound selenium; Se-SELENOP, selenoprotein P-bound selenium; Se-TXNRD, thioredoxin reductase-bound selenium; Se(IV), selenite; Se(VI), selenate.
